# Metabolic potential of endophytic bacteria^☆^

**DOI:** 10.1016/j.copbio.2013.09.012

**Published:** 2014-06

**Authors:** Günter Brader, Stéphane Compant, Birgit Mitter, Friederike Trognitz, Angela Sessitsch

**Affiliations:** AIT Austrian Institute of Technology GmbH, Bioresources Unit, Konrad-Lorenz-Strasse 24, 3430 Tulln, Austria

## Abstract

The bacterial endophytic microbiome promotes plant growth and health and beneficial effects are in many cases mediated and characterized by metabolic interactions. Recent advances have been made in regard to metabolite production by plant microsymbionts showing that they may produce a range of different types of metabolites. These substances play a role in defense and competition, but may also be needed for specific interaction and communication with the plant host. Furthermore, few examples of bilateral metabolite production are known and endophytes may modulate plant metabolite synthesis as well. We have just started to understand such metabolic interactions between plants and endophytes, however, further research is needed to more efficiently make use of beneficial plant-microbe interactions and to reduce pathogen infestation as well as to reveal novel bioactive substances of commercial interest.

## Highlights

**•** Endophytes are a source of a plethora of biologically active substances.
**•** Endophyte-associated metabolites may be needed for the interaction with the plant.
**•** Some metabolites are produced jointly by plant and endophytes.
**•** Endophytes may stimulate or alter metabolite production by the plant.
**•** Metabolite functions include signalling and communication, nutrient acquisition and defense.

## Graphical abstract

**Figure:**
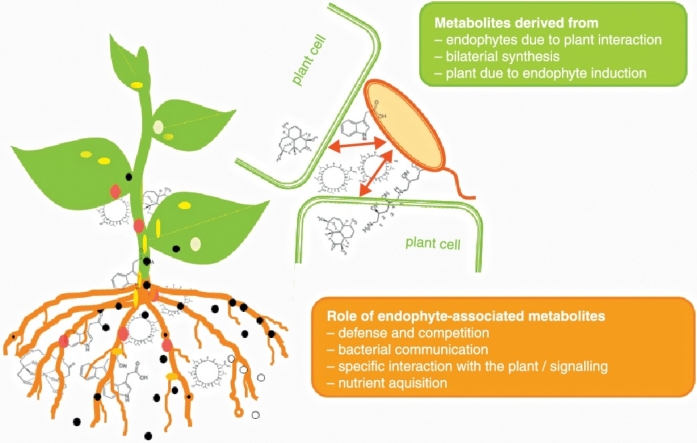

**Current Opinion in Biotechnology** 2014, **27**:30–37

This review comes from a themed issue on **Environmental biotechnology**

Edited by **Hauke Harms** and **Howard Junca**

For a complete overview see the Issue and the Editorial

Available online 22nd October 2013

0958-1669/$ – see front matter, © 2013 The Authors. Published by Elsevier Ltd. All rights reserved.

**http://dx.doi.org/10.1016/j.copbio.2013.09.012**

## Introduction

Living organisms are the source for a vast diversity (>1 million) of different metabolites. The majority of these metabolites have been discovered in plants, but microorganisms are a particular rich source of more than 20 000 biologically active compounds, influencing the performance and survival of other organisms [1]. Of these active compounds the majority are derived from bacteria, mostly from the well investigated genus *Streptomyces* [1,2], which represents the microbial genus most thoroughly investigated for secondary metabolite production. Secondary metabolites are without known specific function in the organisms’ primary metabolism, but their high diversity reflects the biological role particularly in the interactions between organisms in their environment and shows their importance as signals and toxins.

In spite of the decreasing rate of discovery of active metabolites (e.g. antibiotics) in the last decades [3], the genomic revolution of the recent past clearly revealed that our knowledge on the structures and occurrence of metabolites of bacteria is far from saturated. Genome analysis of even well-known bacteria has revealed genes potentially involved in the production of yet unknown metabolites [4,5] and it is assumed that the metabolites identified so far encompass only a small fraction of the existing metabolic repertoire [2]. Another reason why it seems unlikely that the metabolic potential of bacteria is exhaustively known is the fact that so far only a small proportion of bacteria has been cultivated. In soils alone, different studies using DNA:DNA hybridization, Sanger sequencing of clone libraries and next generation sequencing suggest that only a very small percentage of bacteria has been cultivated so far [6]. Moreover, albeit *Actinobacteria*, most prominently the genus *Streptomyces*, proved to be an extremely rich source of secondary metabolites [7,8^•^], the potential of more ‘exotic’ and ‘rare’ actinobacterial taxa is less established [9,10,11] and similar considerations might also hold true for the large fraction of other far less well characterized bacterial taxa. Finally, certain niches, among others the bacteria living in association with plants and in particular inside plants (endophytes), are less well investigated for their metabolic potential than cultivable soil bacteria. Endophytes are also of special interest for their high number of microbial niches and environments they may inhabit and provide therefore a high potential as a less exploited resource. In the current review we understand endophytes as non-phytopathogenic organisms, which colonize plant tissues at least part of their lifetime [12]. Nevertheless, to discuss the potential and function of metabolites we briefly also take into account plant pathogenic microorganisms, which may be very closely related to non-pathogenic species.

## Endophytes as a source of secondary metabolites

Considerable amount of information exists on the metabolic potential of endophytic fungi and exciting possibilities for exploiting endophytic fungi for the production of a plethora of known and novel biologically active secondary metabolites (reviewed by [13,14]). Bacteria can also thrive as endophytes in various plants and plant parts, but are less investigated for their metabolic potential. Various studies have shown that endophytic bacteria may, following rhizosphere soil colonization, be detected inside the endorhiza, in stems, leaves as well as inside plant reproductive organs of different host plants [15,16,17]. Endophytes have to be adapted to the specific plant environment, which they colonize and therefore, the metabolic potential of endophytes is likely to differ from their soil dwelling counterparts. As the resource-rich environment of the rhizosphere is extremely competitive, and bacteria need to survive in a competitor-rich and predator-rich environment, the rhizosphere microflora is likely to produce a rich arsenal of antibiotic and anti-nematodal compounds. In contrast, obligate endophytic bacteria face a lot less competition reflected in a less metabolite-rich arsenal [18], but they may produce other specific metabolites supporting the or needed for the interaction with the host. However, many endophytic bacteria are facultative plant colonizers and have to compete well in the rhizosphere before entering the plant [16] and might be therefore equipped with a rich arsenal of metabolites involved in defense as well as in interaction with the plant. In this context it has to be stated that the term ‘antibiotic’ as ‘defense weapon’ to other microbes may reflect a rather anthropocentric point of view and that the real function of these compounds in nature is not only the antibiotic function, but the compounds may also play a role in intraspecies and interspecies signalling processes [6,19,20,21].

**Figure 1:**
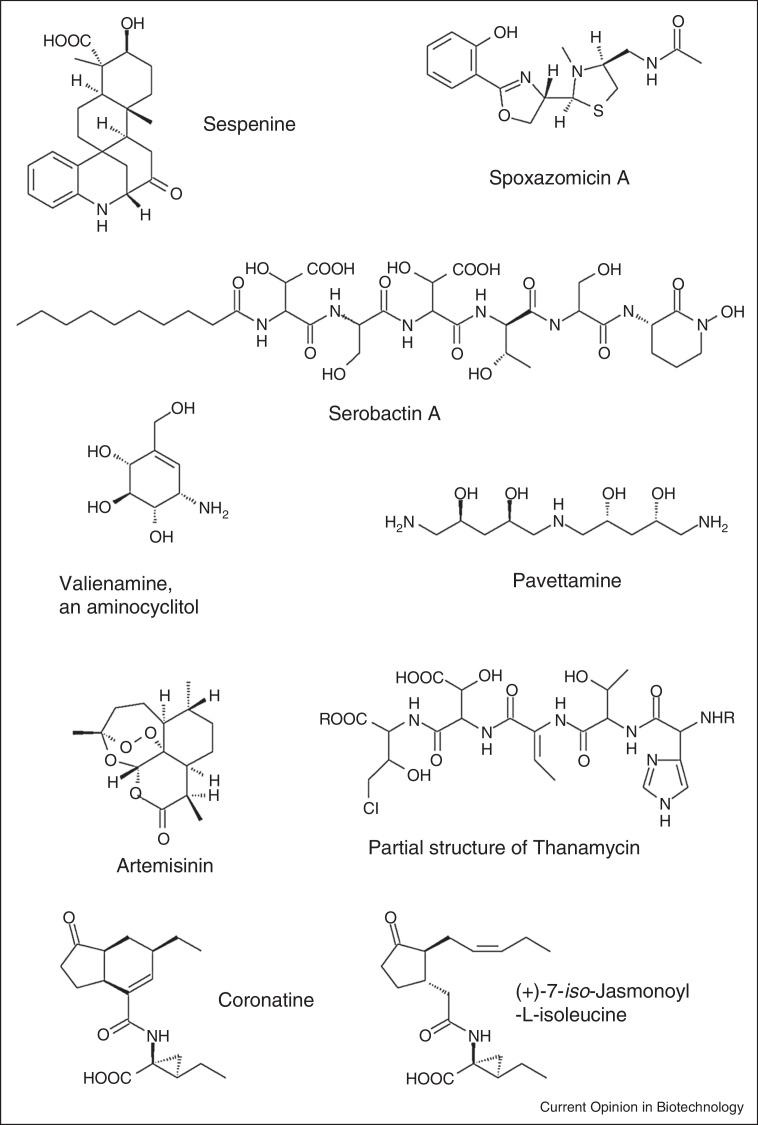
Metabolites of plant associated bacteria. Sespenine is derived from indolosesquiterpenes found in an endophyte (*Streptomyces* sp.) of mangrove trees. Spoxazomicins from an orchid endophyte (*Streptosporangium oxazolinicum*) with structural similarities to pyochelin, a siderophore from *Pseudomonas aeruginosa*, serobactin A, a siderophore from the grass endophyte *Herbaspirillum seropedicae*. Valienamine, as illustration of aminocyclitols, which might be produced by the endophytic *C.* Burkholderia kirkii. Pavettamine is the active toxic principle of South African Rubiaceae, where endophytic *Burkholderia* spp. are crucial for the biosynthesis *in planta*. The partial structure of thanamycine has been elucidated without isolation from bacterial colonies. Coronatine as an example of a plant hormone acting agent from the plant pathogenic *Pseudomonas syringae* and the structure of the actual plant hormone (+)-7-*iso*-jasmonoyl-l-isoleucine.

Many bacteria with the capacity of colonizing plants utilize the nutrient niche of root surfaces in the rhizosphere and most of them might even actively switch from root surface to endophytic lifestyles [15,16]. These bacteria comprise several well characterized species of *Bacillus* and *Pseudomonas* and a number of metabolites, particularly lipopeptides synthesized by non-ribosomal peptide synthesases, have been described to be important for rhizosphere bacteria for antibiosis and for inducing plant defense mechanisms. The structures and functions of *Bacillus* and *Pseudomonas* lipopeptides have been recently thoroughly reviewed (e.g. [6,22,23]). Nevertheless the rich repertoire of metabolites found in endophytic *Actinobacteria* [8^•^] suggests that a large fundus of secondary metabolites produced by endophytic bacteria remains to be discovered. This is underlined by recently described multicyclic indolosesquiterpenes (Figure 1) found in the endophytic *Streptomyces* sp. HKI0595 of the mangrove tree *Kandelia candel* [24], antitrypanosomal alkaloids spoxazomicins A-C (Figure 1) produced by the endophytic actinomycete *Streptosporangium oxazolinicum* K07-0450^T^ found in orchids [25,26] with structural similarities to siderophores from *Pseudomonas aeruginosa* and a series of NRPS (non-ribosomal peptide synthases) and PKS (polyketide synthases) gene clusters with uncharacterized metabolites were found to be produced by endophytes of Chinese medicinal plants [27,28]. The rich metabolic repertoire of endophytic bacteria is also shown in more than 100 actinobacterial isolates found as endophytes in Australian trees [29] and in more than 300 diverse actinobacterial strains found in the medicinal plant *Maytenus austroyunnanensis* [8^•^]. Furthermore, cultivation-independent analysis of bacterial endophytes of Chinese medicinal herbs based on the analysis NRPS and PKS gene fragments suggested the production of so far unknown metabolites [28]. Overall, only a tiny fraction of plant-associated *Actinobacteria* has been described so far representing a promising source of novel secondary metabolites.

## Function of metabolites in plant-bacteria interactions

**Figure 2:**
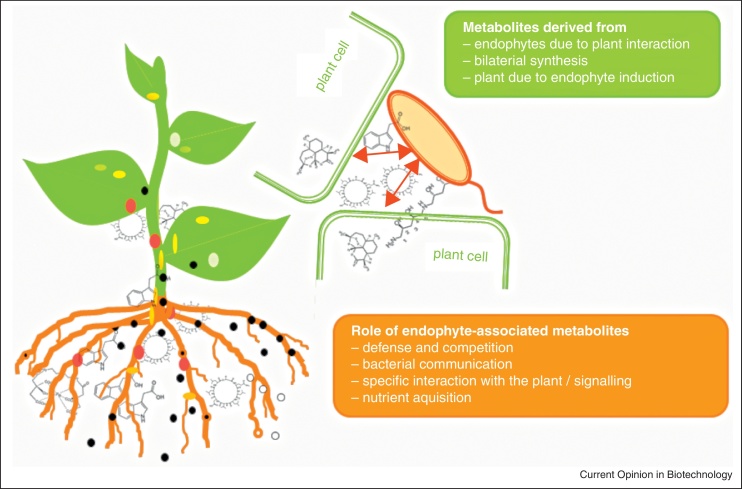
Schematic overview showing the different types of plant-endophyte interactions leading to the synthesis of metabolites, which are in many cases not produced by the macro- or microsymbiont alone or in different quantities. Furthermore, the different known functions of endophyte-associated metabolites are presented.

Many bacteria closely interacting with plants produce secondary metabolites as agents needed for nutrient uptake (for a schematic overview see Figure 2), in particular siderophores involved in iron acquisition (reviewed by [30]). Recently, in the diazotrophic endophyte *Herbaspirillum seropedicae* colonizing many grass crops, the structures of the amphiphilic lipopeptides serobactin A, B and C produced by NRPS (Figure 1) acting as siderophores have been described [31]. Moreover, metabolites acting as agents in biofilm formation and as toxins, virulence factors [6] or interfering with hormone signalling in plants [32,33] have been reported. The latter functions may be also important for plant pathogens. Generally, the boundary between pathogens and endophytes or phytohormones and toxins are not always clear-cut and especially hormone production is a widely spread characteristic of phytopathogens and plant growth-promoting bacteria (for a review see [32]). Plants produce several classes of phytohormones including auxins, cytokinins, brassinosteroids, gibberellins, abscisic acid, ethylene, jasmonates and strigolactones playing roles in development and stress responses. Cross talk and fine tuning of the different phytohormone pathways is essential for plant development, stress and defense responses ([34]; reviewed by [35]) and associated bacteria can interfere with plant signalling. In beneficial bacterial endophyte — plant interactions the production and modulation of auxins and ethylene play an essential role in plant development [32,36], but also stress (e.g*.* drought) tolerance has been reported to be influenced by endophyte-derived hormones. For example, abscisic acid and gibberellins produced by the endophyte *Azospirillum lipoferum* have been shown to be involved in alleviating drought stress symptoms in maize [37]. Interestingly, plant-associated bacteria do not only produce genuine plant hormones but also compounds mimicking the effect of the natural plant hormones as structural analogues (Figure 1). This is the case for coronatine produced by several plant pathogenic *Pseudomonas* species mimicking the active natural (+)-7-*iso*-jasmonoyl-l-isoleucine [38]. Coronatine acts as very active jasmonate finally showing phytotoxicity [33] and plays a role in suppressing stomatal closure and defense responses [39]. It will be interesting to see if plant hormone mimicry encoded by NRPS and PKS gene clusters with so far unknown function is a common feature in plant-associated bacteria.

## Adaptation to endophytic lifestyle and the potential to produce secondary metabolites

**Figure 3:**
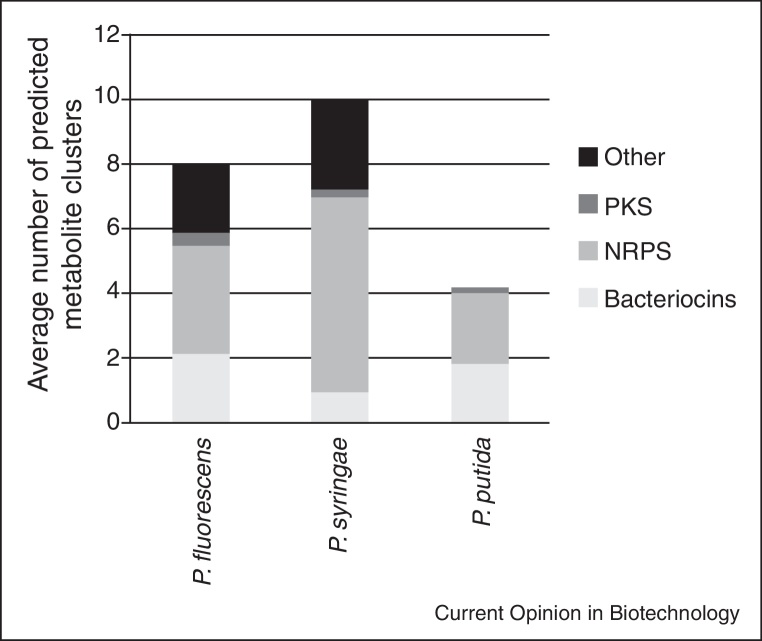
Average numbers of metabolite gene clusters predicted by antiSmash 2.0 [40^••^]. The numbers are the mean of 6 *Pseudomonas fluorescens* (plant-associated) strains, 5 *P. syringae* (plant pathogens) and 9 *P. putida* strains (no association with plants) and contain all fully sequenced and published genomes in the given category.

When comparing the amounts of predicted secondary metabolites of all completely sequenced *Pseudomonas* strains by antiSmash [40^••^], a prediction software for secondary metabolite production, it can be seen that pseudomonads associated with eukaryotes as plant pathogens (*P. syringae*) or as endophytes or epiphytes (*P. fluorescens*) host a higher number of gene clusters encoding for secondary metabolites, in particular NRPS and other metabolites (predicted quorum sensing signals, not further characterized metabolites) than free living *P. putida* strains (Figure 3). In the latter strains the number of bacteriocins potentially involved in competition with closely related species is higher [41]. It might be that the plant-associated lifestyle requires adaptation to several niches, in which different metabolites are required. On the other hand, specialized endophytes such as obligate endophytes or endophytes colonizing only specific niches may produce a lower number of potential secondary metabolites. Metabolites furthermore act as signals for interaction (communication) with the plant and host-specific signal exchange may occur as reported for plant–fungal interactions [42].

Although genome reduction is a general mechanism of (intracellular) pathogens [43], and also highly adapted symbiotic and obligate endophytic bacteria like *Candidatus* Burkholderia kirkii show clear reduction of their genome compared to free living relatives [44^•^], this does not necessarily lead to a complete loss of the potential to produce secondary metabolites. Quite on the contrary, it has been speculated that *C.* Burkholderia kirkii produces metabolites to protect its host plant *Psychotria kirkii* (Rubiaceae) against pathogens or herbivores. The genome of *C*. Burkholderia kirkii contains several biosynthetic genes responsible for secondary metabolite production and especially two clusters for the biosynthesis and transport of sugar analogues of the C7N family of aminocyclitols (Figure 1). Several members of the C7N aminocyclitols display antibiotic, antifungal or insecticidal activity [45]. Several *Psychotria* species harbor *Burkholderia* spp. within specialized leaf nodules. It remains to be seen how far these endophytes contribute to the metabolic potential of *Psychotria*. Such specialized endophytes may play a role in plant defense by producing toxins active against herbivores as it is well known for endophytic fungi, especially for the genera *Epichloe* and *Neotyphodium* (see e.g*.* [13,46]). Also, bacteria living in association with marine eukaryotes are made responsible for the production of various toxins involved in the defense mechanism of the eukaryotic host, which include dinoflagellates and tunnicates [47]. Interestingly, the saprotrophic fungus *Rhizopus microsporus* produces rhizoxin and is responsible for rice seedling blight, but the actual producer of the toxin is the bacterium *Burkholderia endofungorum* [48–50]. It is remarkable that related *Burkholderia* spp. live in close association with *Psychotria* plants and it remains to be elucidated if plant toxin production might be in more cases related to bacterial endophytes. Other Rubiaceae plants, namely *Fadogia*, *Pavetta* and *Vangueria*, which can all cause a disease (called ‘gousiekte’) in ruminants feeding on these plants host different *Burkholderia* spp. suspected to play a role in production of the toxin causing the disease, the polyamine pavettamine (Figure 1) [51,52^•^].

## Endophytes as contributors to plant metabolite production

So far we discussed the direct metabolic potential of endophytic bacteria. However, two other indirect ways exist, how endophytic bacteria play a role for the metabolic potential of plants (for a schematic overview see Figure 2). First, bacterial endophytes may strongly influence the performance, growth and stress tolerance of plants [16,53,54]. In this respect, it is remarkable that an endophytic actinobacterium, *Pseudonocardia* sp. strain YIM 63111, is able to enhance the production of the antimalarial compound artemisinin (Figure 1) in its host plant *Artemisia annua* [55^•^]. The induction of secondary metabolite production by endophytes might be a much more widespread phenomenon in aromatic and medicinal plants. Second, some metabolites are not only produced by a single organism, but might be produced by a plant in combination with associated bacteria. This has been discussed for the flavour of strawberries, where furanoids are responsible for the typical fragrant [56] and where it has been shown that plant-associated methylobacteria influence the quality and quantity of the flavour [57]. Also, for the biosynthesis of the polyamine pavettamine (Figure 1) of South African Rubiaceae, it has been discussed that the production might be because of bilateral biosynthesis as only nodulating plants produce the toxin. Furthermore, nodulating plants void of pavettamine production have been found and plant cell cultures without bacteria do not produce pavettamine but more common polyamines [58^•^].

## Detection of metabolites in plant association

The majority of metabolites from endophytic bacteria have been characterized after isolating bacteria and growing them *in vitro*. Novel developments in the *in situ* analysis of metabolites might provide new opportunities to detect and to describe also metabolites specifically produced during the interaction with living plants. The overall concentration of compounds produced by plant-associated bacteria in roots and the rhizosphere is usually low (usually < 10 μg/g), making the direct structure elucidation challenging. Direct analysis of metabolites *in situ* has been achieved for antibiotic lipopeptides from several *Bacillus subtilis* and for pyrrolnitrin, 2,4-diacetylphloroglucinol and phenazine-1-carboxylic acid from *Pseudomonas fluorescens* strains [6]. Local concentrations might be still higher and biosensor-based approaches might be important tools to detect various metabolites *in situ* such as for *Pseudomonas fluorescens* CHA0 lipopeptides [59], but the detection of unknown compounds remains challenging, and for a quantitative approach mainly LC–MS based methods have been successful [6]. Apart from difficulties in detecting unknown compounds also the composition of already described metabolites may vary significantly *in vitro* and *in planta*. For example, the comparison of metabolic profiles produced in growth medium and *in planta* showed clear differentiation of lipopeptides produced by *Bacillus amyloliquefaciens* S499 with iturin and fengycin underrepresented in the root samples, while surfactins were stronger accumulated in roots. Combined electrospray and imaging mass spectrometry-based approaches were used to determine the detailed pattern of surfactins, iturins and fengycins [60]. Novel metabolites only produced in specific niches or low concentrations within the plant are not easily found, albeit genomic analysis can point to potential genes and help in the prediction of those metabolites. A breakthrough has been achieved here with the description of thanamycin (Figure 1) [61^••^]. After applying PhyloChip-based analysis secondary metabolites synthetized by a NRPS of *Pseudomonas* sp. strain SH-C52 were identified to be involved in suppressing sugar beet diseases caused by *Rhizoctonia solani* [62^•^]. On the basis of this discovery Watrous *et al.* [61^••^] could establish the partial structure of such a metabolite, thanamycin, with nanospray desorption electrospray ionization mass spectrometry (nanoDESI MS) combined with MS data alignment and molecular networking. This technology allowed the direct analysis and partial structure elucidation of a chlorinated lipopeptide thanamycin on Petri dishes without any sample preparation. NanoDESI MS or related technologies might in future even allow the detection of novel metabolites directly in environmental or root samples [63]. For example, MALDI-FTICR MS imaging has shown the production of fusaricidin lipopetides of *Paenibacillus polymyxa* in interaction with *Fusarium oxysporum* on plate [64]. These non-invasive methods have also the additional advantage to allow time-course metabolic analysis and can represent effective tools for the analysis of intermediate steps including less stable compounds of biosynthetic pathways [63–66].

## Conclusions

Evidence is increasing that endophytic bacteria have a high potential in producing a wide range of so far undescribed metabolites. Partly, the concentration and circumstances under which these metabolites are produced are not well understood but the genomic revolution together with the steady development of analytic techniques will certainly accelerate the discovery of such cryptic compounds and the future will show how many novel chemical structures and compounds are encoded in endophytic bacterial genomes. Moreover, other known (plant) metabolites might turn out to be partly or fully derived from endophytic or associated bacterial metabolism. It remains to be seen how widespread this phenomenon might be or if it is restricted to genera of the Rubiaceae and certain fragrants. An additional challenge in future research is the detection and characterization of metabolites formed in niches on and in plants or under specific circumstances under natural conditions only, as indicated by the only partial realization of the metabolic potential of bacteria grown *in vitro*.

## References and recommended reading

Papers of particular interest, published within the period of review, have been highlighted as:

• of special interest
•• of outstanding interest
